# Amidinoquinoxaline-Based Nitrones as Lipophilic Antioxidants

**DOI:** 10.3390/antiox10081185

**Published:** 2021-07-26

**Authors:** Nadia Gruber, Liliana Orelli, Cristina Minnelli, Luca Mangano, Emiliano Laudadio, Giovanna Mobbili, Pierluigi Stipa

**Affiliations:** 1 Química Ogánica II, Departamento de Ciencias Químicas, Facultad de Farmacia y Bioquímica, Universidad de Buenos Aires, CONICET, Junín 956, Buenos Aires 1113, Argentina; ngruber@ffyb.uba.ar (N.G.); lorelli@ffyb.uba.ar (L.O.); 2 Dipartimento di Scienze della Vita e dell’Ambiente (DISVA), Università Politecnica delle Marche, via Brecce Bianche, 60131 Ancona, Italy; c.minnelli@staff.univpm.it; 3 F. Hoffmann-La Roche AG, Grenzacherstrasse 124, 4070 Basel, Switzerland; luca.mangano01@universitadipavia.it; 4 Dipartimento di Scienze e Ingegneria della Materia, dell’Ambiente ed Urbanistica (SIMAU), Università Politecnica delle Marche, via Brecce Bianche, 60131 Ancona, Italy; e.laudadio@staff.univpm.it

**Keywords:** antioxidants, nitrones, spin trapping, reactive oxygen species, density functional theory (DFT), free radicals

## Abstract

The potential of nitrones (N-oxides) as therapeutic antioxidants is due to their ability to counteract oxidative stress, mainly attributed to their action as radical scavengers toward C- and O-centered radicals. Among them, nitrones from the amidinoquinoxaline series resulted in interesting derivatives, due to the ease with which it is possible to introduce proper substituents within their structure in order to modulate their lipophilicity. The goal is to obtain lipophilic antioxidants that are able to interact with cell membranes and, at the same time, enough hydrophilic to neutralize those radicals present in a water compartment. In this work, the antioxidant efficacy of a series of amidinoquinoxaline nitrones has been evaluated regarding the oxidation of 2-deoxyribose and lipid peroxidation. The results have been rationalized on the basis of the different possible mechanisms involved, depending on some of their properties, such as lipophilicity, the ability to scavenge free radicals, and to undergo single electron transfer (SET) reactions.

## 1. Introduction

It has already been well documented that peroxidative processes, originating from free radicals, are involved in various human disease states, such as, for example, inflammation, carcinogenesis, ischemia/reperfusion injury [1] and aging [2].

**Figure 1:**
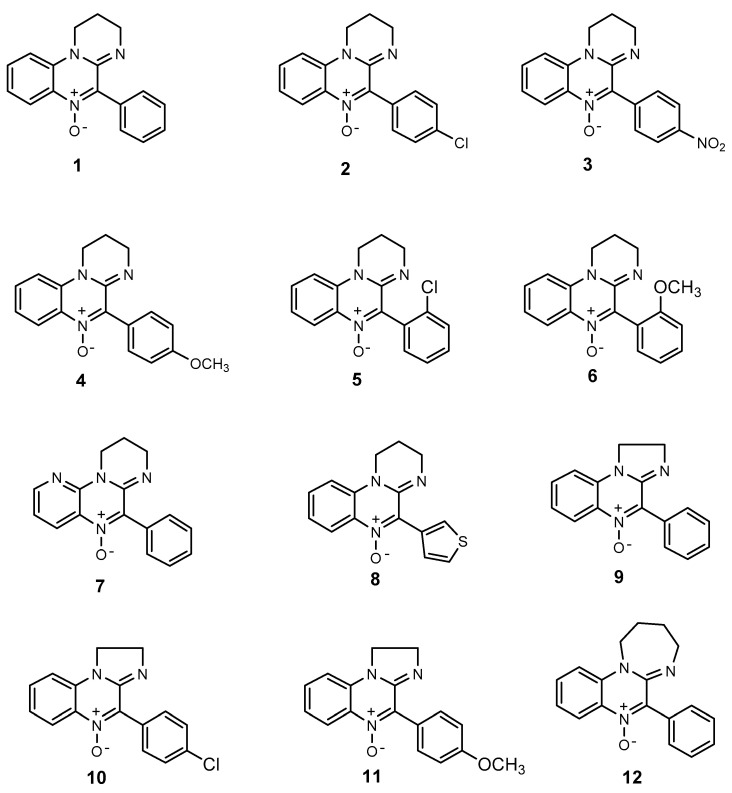
Structure of amidinoquinoxaline-based nitrones.

As a consequence, a large number of these states have been recently ascribed to the “unbalanced” production of mainly O-centered radicals (reactive oxygen species (ROS)) but also of N-centered ones (reactive nitrogen species (RNS)) [3]. These species are normally produced by cells to establish a kind of homeostasis [4], but in some circumstances they may induce stress, leading to the interruption of some cellular functions. Such an “oxidative stress” is characterized by enhanced production of ROS, with the simultaneous impairment of cellular defense mechanisms [5]. The process usually starts with a one-electron reduction of molecular oxygen, likely taking place in mitochondria, to form the superoxide radical anion O_2_**^●−^** which, in turn, may give rise to Hydroperoxyl (HOO**^●^**) as well as hydroxyl radicals (HO**^●^**). These species are viewed as the main cause of “oxidative stress” because they are able to abstract hydrogen atoms from different substrates, producing C-centered radicals, which in turn react with molecular oxygen to yield other ROS. For these reasons, the study of antioxidant derivatives that are able to efficiently hamper these species’ diffusion is continuously in progress. Within this field, nitrones (N-oxides) represent an interesting family of derivatives that have been widely exploited in EPR spin trapping since the end of the 1960s [6,7,8] because they act as efficient radical scavengers [9,10]; therefore, they could also play an important role as chain-breaking antioxidants [11], since they efficiently react with both C-centered and O-centered radicals. Their structure can be modified by introducing different functional groups in order to improve the corresponding spin adduct stability [12,13,14,15,16], as well as modulating their lipophilicity to obtain a more efficient antioxidant in a biological environment [4,5]. The behavior of nitrones as spin traps from the amidinoquinoxaline [17,18] and benzoxazine series [19] has been previously studied by our research groups; since the latter also resulted in efficient antioxidants [20], we were prompted to extend the investigation to their amidinoquinoxaline analogs. Hence, in the present study, the antioxidant efficacy of some amidinoquinoxaline derivatives (Figure 1) has been evaluated toward the oxidation of 2-deoxyribose and lipid peroxidation, studied in liposomes employed as a model membrane. The corresponding results have been explained on the basis of the different possible mechanisms involved, depending on some of their properties. For example, their lipophilicity has been evaluated by determining their oil/water partition coefficient, while the ability to scavenge free radicals and undergo single electron transfer (SET) reactions was investigated by means of proper density functional theory (DFT) calculations.

## 2. Materials and Methods

Reagents, solvents, and starting materials were purchased from standard sources and were used without any further purification. Melting points were determined on a Thomas Hoover capillary apparatus and are uncorrected. ^1^H and ^13^C NMR spectra were recorded on a Bruker Bio Spin Avance III 600 MHz spectrometer, using deuterochloroform as the solvent. Chemical shifts are reported in parts per million (ppm), relative to TMS as an internal standard. Coupling constants are reported in Hz. D_2_O was employed to confirm exchangeable protons (ex). Splitting multiplicities are reported as singlet (s), broad signal (bs), doublet (d), double doublet (dd), and multiplet (m). HRMS (ESI) was performed with a Bruker MicroTOF-Q II spectrometer.

### 2.1. Synthesis of Amidinoquinoxaline N-Oxides 1–12

Amidinoquinoxaline N-oxides were synthesized according to the method previously reported by our group [21], which includes the cyclodehydration of aminoamides. Compounds 1–4 [21], 5 [22], 6–8 [17], 9 [23] and 11 [18] are described in the literature. Yields and analytical data of nitrone 12 and the corresponding aminoamide N-(4-(2-nitrophenylamino)butyl)-2-phenylacetamide are as follows.

#### 2.1.1. 6-Phenyl-8,9,10,11-tetrahydro-[1,3]diazepino [1,2-a]quinoxaline 5-oxide (12) 

This compound was obtained as a yellow solid (8% yield), mp = 183–184 °C (from EtOH). ^1^H NMR (600 MHz, CDCl_3_, 25 °C, TMS): δ = 8.32 (1H, d, J = 8.2), 7.67 (2H, d, J = 7.7), 7.44–7.51 (3H, m), 7.39–7,43 (1H, m), 7.13–7,18 (1H, m), 7.08 (1H, d, J = 8.3), 4.08 (2H, bs), 3.88 (2H, bs), 2.16–2.22 (2H, m, bs), 1.99–2.05 (2H, m, bs). ^13^C NMR (151 MHz, CDCl_3_, 25 °C): * δ = 148.1, 141.3, 136.5, 131.5, 131.4, 130.5, 129.5, 128.1, 121.7, 121.2, 112.3, 49.7, 48.6, 25.5, 24.4. HRMS (ESI) *m/z*: [M + H]^+^ calculated for C_18_H_18_N_3_O: 292.1444. Found: 292.1453. * Overlapping signals.

#### 2.1.2. N-(4-(2-Nitrophenylamino)butyl)-2-phenylacetamide

This compound was obtained as a yellow solid (91% yield), mp = 89–91 °C (from hexane/chloroform). ^1^H NMR (600 MHz, CDC_l_3, 25 °C, TMS): δ = 8.15 (1H, dd, J = 8.6, 1.4 Hz), 7.98 (1H, bs ex), 7.40–7.44 (1H, m), 7.32–7.36 (2H, m), 7.26–7.30 (1H, m), 7.23–7.26 (2H, m), 6.80 (1H, d, J = 8.6 Hz), 6.62–6.65 (1H, m), 5.49 (1H, bs ex) 3.57 (2H, s), 3.25–3.30 (4H, m), 1.63–1.69 (2H, m), 1.61–1.55 (2H, m).^13^C NMR (151 MHz, CDCl3, 25 °C) δ = 171.2, 145.5, 136.4, 135.0, 132.0, 129.5, 129.2, 127.5, 127.0, 115.4, 113.8, 44.0, 42.6, 39.2, 27.3, 26.3. HRMS (ESI) *m/z*: [M + H]^+^ calculated for C_18_H_22_N_3_O_3_: 328.1656. Found: 328.1645.

### 2.2. Log P and Antioxidant Activity Determinations

Egg-yolk L-α-phosphatidylcholine (Egg-PC) (PC), 2-deoxyribose (2-DR), phenyl-N-tert-butylnitrone (PBN), 2,2’-azobis(2-amidinopropane) dihydrochloride (AAPH), 1-octanol and all other reagents and solvents were purchased from Sigma Aldrich (St. Louis, MO, USA) and used without further purification.

Spectrophotometric measurements were recorded on a microplate reader (Synergy HT MicroPlate Reader Spectrophotometer, BioTek Instruments, Inc., Winooski, VT, USA). 

All the experiments were run in duplicate and were repeated at least 4 times.

### 2.3. Antioxidant Activity Determinations by TBARS Assay

The antioxidant activity was studied by TBARS assay (thiobarbituric acid reactive substances) using the peroxidation of L-α-phosphatidylcholine (PC) liposome and oxidation of 2-deoxyribose (2-DR) models, as previously described [24,25]. At the end of the experiments, the absorbance was recorded at 532 nm to determine the aldehydic breakdown products of oxidation. The antioxidant activity of the studied nitrones was expressed as % inhibition, according to the following equation:

% Inhibition = (1 − ΔA_treated_/ΔA_untreated_) × 100, (1)

where ΔA_treated_ is the difference of the absorbance between the oxidized and non-oxidized PC and 2-DR (treated with antioxidant), while ΔA_untreated_ is referred to ΔAPC and ΔADR for the peroxidation of PC liposome and oxidation of 2-DR, respectively, and represent the difference of the absorbance between the oxidized and non-oxidized control (without antioxidant).

#### 2.3.1. Peroxidation of L-α-Phosphatidylcholine Liposome

L-α-phosphatidylcholine liposomes with (treated) and without (untreated) antioxidants were prepared by the “thin film hydration” method. An appropriate amount of L-α-phosphatidylcholine in chloroform and antioxidant nitrones was mixed to obtain a molar ratio between nitrones and lipid of 1:50. The solvent was removed under reduced pressure and dried for 2 h. The obtained thin film was then hydrated in PBS (5 mM, pH 7.4) to obtain final lipid and antioxidant concentrations of 2.5 mM and 0.05 mM, respectively. After 24 h of incubation, the resulting MLV (multilamellar vesicles) were sonicated for 12 min using a Sonic Vibracell sonicator (20 sec on; 20 sec off, 40%) at 0 °C to obtain SUV (small unilamellar vesicles). The mean diameter of liposomes, determined by dynamic light scattering (DLS) using Malvern Instruments (GmbH, Marie-Curie-Straße 4/1, 71,083 Herrenberg, Germania), was found to be in the range of 90–110 nm. Oxidized samples (treated or untreated with nitrones) were obtained by adding, to each dispersion (300 µL), 25 µL AAPH 65 mM (5 mM final concentration), then incubating for 2 h at 310 K, followed by the addition of 10 µL of 20 mM methanolic BHT. For non-oxidized samples, PBS was added instead of AAPH. Then, 0.9 mL of TBA-TCA-HCl [0.375% *w/v* TBA (thiobarbituric acid), 15% *w/v* TCA (trichloroacetic acid), and 0.2 M HCl] were added and the samples were heated for 15 min at 368 K, followed by cooling and centrifugation at 2200× *g* for 10 min. 

#### 2.3.2. Oxidation of 2-Deoxyribose

In the oxidation of 2-deoxyribose (2-DR) assay, nitrones solutions were prepared in CH_3_CN. Firstly, 2-deoxyribose, EDTA and H_2_O_2_ were dissolved in 50 mM PBS pH 7.4. Then, [Fe(NH_4_)_2_(SO_4_)_2_∙6H_2_O] was dissolved in H_2_O mQ. Briefly, 2.8 mM 2-DR, was incubated for 1 h at 310 K with 0.07 mM [Fe(NH_4_)_2_(SO_4_)_2_∙6H_2_O], 0.035 mM EDTA and 1.4 mM H_2_O_2_ in 50 mM PB (pH 7.4), in the absence or presence of nitrone (5 μM). To prevent the oxidation, also in the absence of AAPH, 10 μL of 10 mM BHT were added to all non-oxidized samples and incubated at 310 K for the same duration. At the end of the incubation period, 10 μL of 10 mM BHT were added to each oxidized sample, followed by 1 mL of 1% (*w*/*v*) TBA in 50 mM NaOH and 1 mL 2.8% (*w*/*v*) TCA. After incubation at 368 K for 30 min, the % inhibition was calculated, as previously described. 

### 2.4. Log P Determination

Solutions of each nitrone were prepared in 1-octanol, and UV−vis spectra were acquired to determine the λmax and the necessary dilutions to obtain absorption values between 0.6 and 1. After having placed 5 mL of each solution in 15-mL conical centrifuge tubes, PBS was added (5 mL, pH 7.4) and the biphasic system was vigorously mixed using a vortex mixer (VELP Scientifica, Usmate Velate MB, Italy) for 4 min. After centrifugation (2000× *g*, 5 min), the octanol phase absorption was measured at λmax; the procedure was repeated to acquire triplicate data. The partition coefficient K_o/w_ is calculated according to the following equation:

K_o/w_ = [nitrone]_oct_/[nitrone]_H2O_ = A_f_/A_i_ − A_f_ (2)

where A_i_ is the initial measured absorbance in octanol, pre-extraction, and A_f_ is the final measured absorbance in octanol, post-extraction.

### 2.5. Density Functional Theory (DFT) Calculations

Density Functional Theory (DFT) calculations were carried out using the GAUSSIAN 09 suite of programs (http://gaussian.com/g09citation/, accessed on 30 June 2021), taking advantage of the resources available at Cineca Supercomputing Center (http://www.cineca.it/HPSystems, 30 June 2021). All calculations on paramagnetic species were carried out with unrestricted formalism, giving S^2^ = 0.7501 ± 0.0003 for spin contamination (after annihilation). Thermodynamic quantities were computed in vacuo at 298 K by means of frequency calculations that were performed by employing the B3-LYP functional in conjunction with the 6-31+G(d,p) basis set, starting from molecular geometries computed at the B3-LYP/6-31G(d) level of theory. In the frequency calculations, negative values (imaginary frequencies) have never been found, demonstrating that all quantities were referred to as geometry minima.

### 2.6. Statistical Analyses 

Data are presented as mean ± S.D. Statistical comparison of differences among nitrones derivative and PBN nitrone data was carried out using Student’s *t*-test. Values of *p* < 0.05 were considered statistically significant.

## 3. Results and Discussion

The antioxidant activity of amidinoquinoxaline N-oxides was evaluated in PC liposomes by measuring the percentage inhibition of aldehydic breakdown products (TBARS) produced during AAPH-induced lipid peroxidation, using the TBA assay. 

**Figure 2:**
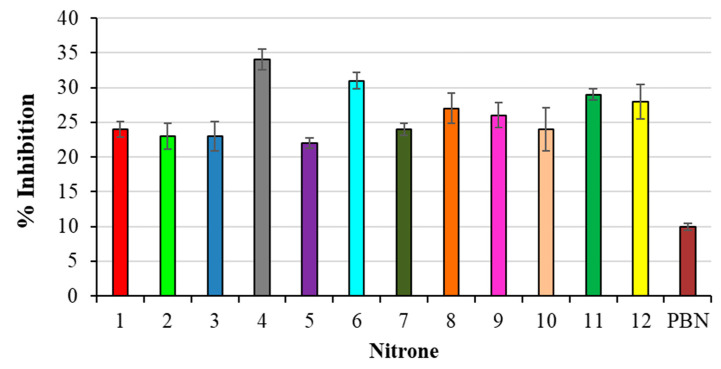
% Inhibition of TBARS formation in AAPH-induced liposomes peroxidation. Results are expressed as mean ± SD (*n* = 5). All nitrones derivatives are significant against PBN nitrone.

All nitrones that were studied showed higher antioxidant activity in our lipid peroxidation experiments than PBN as a reference, as shown in Figure 2.

In particular, the best results have been obtained with compounds 4, 6 and 11, all bearing a methoxy substituent at the aromatic ring, present at the α-Carbon, with respect to the Nitrone N-O group. On the other hand, the less active derivatives of the series were those bearing an electron-withdrawing group (-Cl; -NO_2_) in the same position (2, 3, 5, 10). 

**Figure 3:**
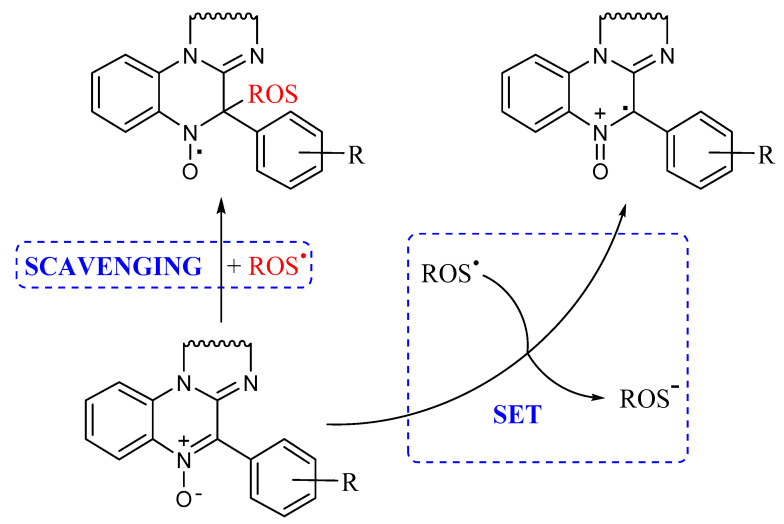
**A** possible mechanism for nitrone antioxidant activity.

As already mentioned, a possible mechanism by which nitrones can act as antioxidants is represented by their radical scavenging activity, well exploited in their use in EPR spin trapping experiments, producing the corresponding persistent nitroxide as a reaction product, as shown in Figure 3. However, when carrying out our lipid peroxidation experiments in the cavity of an EPR spectrometer, a signal attributable to a nitroxide has never been detected. Another possible mechanism is represented by a single electron transfer (SET) process between nitrone and oxygen-centered radicals to yield the corresponding radical cation and anion, respectively, as reported in Figure 3.

Such behavior depends on the redox potential of the species involved; concerning the nitrones under investigation, their tendency to undergo oxidation should be lowered by the presence of electron-withdrawing groups. This seems to be in line with our findings, indicating that derivatives 2, 3, 5 and 10 are identified as being less active as antioxidants.

**Figure 4:**
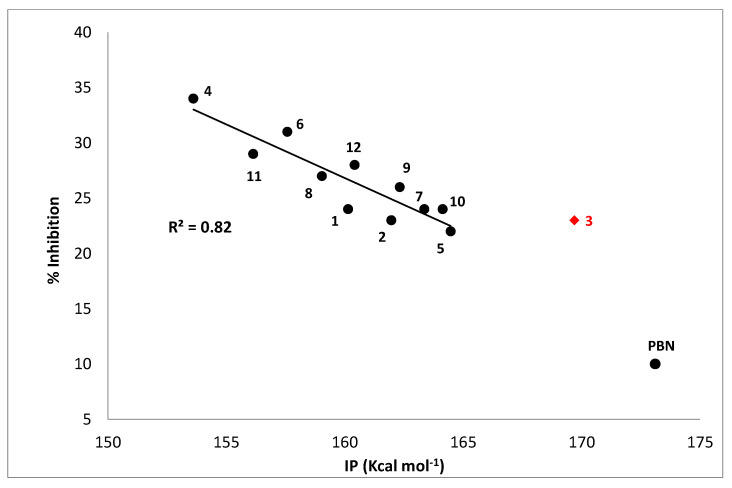
% Inhibition of TBARS formation in the AAPH-liposome peroxidation system vs. calculated ionization potential (IP, kcal mol^−1^). Compound 3 was excluded, since it shows different behavior, maybe due to the presence of the nitro group in its structure. PBN was included for comparison.

In order to have additional indications concerning this possibility, the ionization potentials of all studied nitrones have been computed by means of proper DFT calculations in the gas phase, and the results have been related to their antioxidant activity, expressed as the percentage of inhibition of TBARS formation in AAPH-induced liposome peroxidation. The resulting plot is shown in Figure 4 and reveals a satisfactory correlation between the % inhibition and IP, with the exception of derivative 3, the only nitrone bearing a NO_2_ substituent. Such a behavior let us surmise that, in these experimental conditions, a SET mechanism likely represents the main operating one, according to what was previously hypothesized for liponitroxides in membranes [26].

**Figure 5:**
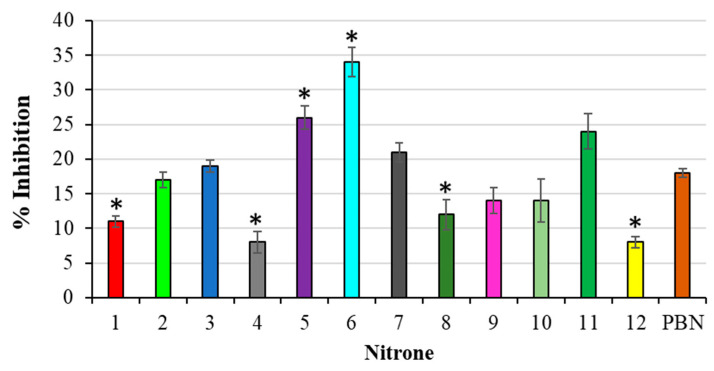
% Inhibition of TBARS formation during deoxyribose oxidation. Results are expressed as mean ± SD (*n* = 5). Nitrones derivative versus PBN nitrone, * *p* < 0.05.

The antioxidant activity of the nitrones was also investigated by means of the 2-deoxyribose assay (DOX). The experimental results, expressed as the % inhibition, are reported in Figure 5.

In this case, many of the derivatives under investigation proved less active with respect to what was previously found in the lipid peroxidation assay, and less active than PBN, as well. In addition, since it was impossible to achieve a correlation between the % inhibitions and nitrones IPs, the predominance of a SET mechanism, in this case, should be ruled out. Considering that the 2-deoxyribose assay is carried out in an aqueous medium, and that HO**^●^** radicals are produced, we believe that, in these conditions, the nitrone antioxidant activity can mainly be ascribed to a combination of different factors, among them, their water solubility, and their ability to scavenge HO**^●^** radicals could play a determining role.

In order to evaluate such an assumption, a multiparametric approach has been implemented, where relevant parameters of the nitrones have been included (Table 1). Among them, the n-octanol-water partition coefficient (K_o/w_) was experimentally determined, showing a clear dependence on the ring size. In fact, five-membered amidine rings are considerably less water-soluble than 6- and 7-membered ones, which can be ascribed to the differences in the corresponding amidine nitrogen atoms. The dipolar moment (μ), IP, the chosen dihedral angle and MP2 charges were instead obtained in silico, using proper calculations. The chosen dihedral angle involves the nitrone moiety and the α-aryl substituent, which, together with the MP2 charges in the α-carbon, play a determining role in the spin trapping properties of these nitrones [17].

**Figure 6:**
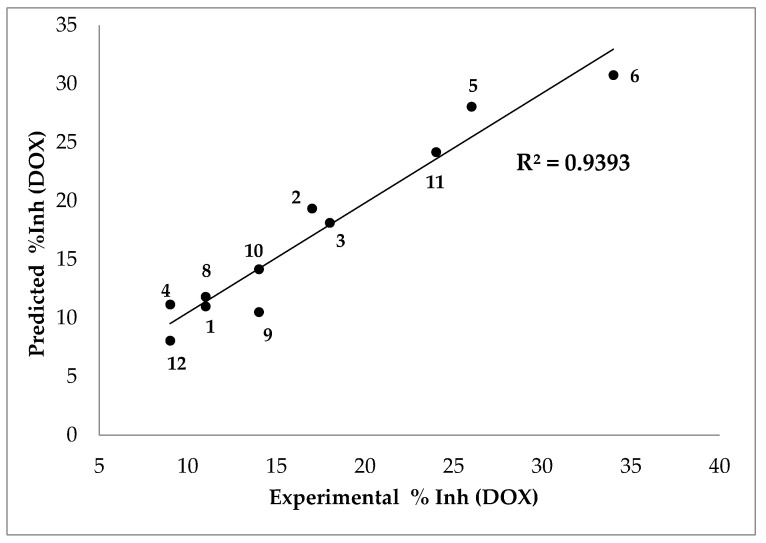
Predicted vs. experimental percentage inhibitions results in DOX assay. Compound 7 was excluded from the analysis (see text).

The following equation predicts the % inhibition results in a DOX assay with a very good linear correlation with the experimental results (Figure 6):

(3) $$\%\mathrm{Inh}\;\left(\mathrm{DOX}\right)=1.29802K_{o/w}-1.13928\;\delta +235.581\mathrm{MP}2+2.88921\;\mu -1.36924\;\mathrm{IP}+129.467$$

where %Inh (DOX) is the inhibition percentage by 2-deoxyribose assay, K_o/w_ is the n-octanol-water partition coefficient, δ the dihedral angle, MP2 the charge, μ the dipolar moment, and IP the ionization potential.

This equation was obtained by excluding compound 7 from the analysis, as its result was unsuitable, likely because the presence of a nitrogen atom in its fused aromatic ring significantly modifies the electron density distribution, affecting its chemical behavior [13].

The corresponding relevance of each coefficient in the equation, provided by a Molegro virtual docker, is shown in Table 2. The interpretation of these data suggests the coexistence of both types of antioxidant mechanisms, i.e., radical scavenging and SET. The dihedral angle appears as the main contributing factor, which, in accordance with our previous spin trapping studies [17] is related to the stability of the spin adduct. We found that the more “twisted” the nitrones, depending on the steric hindrance of the substituent at the nitrone α-carbon, the more persistent the corresponding spin adduct.

## 4. Conclusions

Amidinoquinoxaline nitrones represent an interesting class of lipophilic antioxidants, due to the ease with which it is possible to introduce proper substituents within their structure, in order to modulate their oil/water partition coefficient. In this work, the antioxidant efficacy of a series of these derivatives has been evaluated regarding the oxidation of 2-deoxyribose and lipid peroxidation. In order to explain the obtained results, a wider point of view has been adopted. We have considered their lipophilicity via the experimental evaluation of the oil/water partition coefficient, mainly governing the distribution within lipid membranes. In addition, by means of proper DFT calculations, their attitude to adding free radicals at the nitrone C=N double bond acting as spin traps, as well as their tendency to act as reducing agents in single electron transfer (SET) reactions, have been considered. From this study, it emerged that, concerning lipid peroxidation, the antioxidant activity of these derivatives correlates well with the corresponding ionization potential, taken as a reference for a possible SET process. Regarding 2-deoxyribose oxidation inhibition, different mechanisms could be operating at the same time, as demonstrated by the multivariable analysis we carried out, allowing us to obtain a relationship able to predict the antioxidant behavior in such a milieu.

## Figures and Tables

**Table 1:** Nitrone parameters used for multivariable analysis.

Comp.	K_O/W_	Dihedral Angle (δ, °)	MP2 Charge	Dipolar Moment (μ)	IP (Kcal/mol)	DOX Inhibition (%)
1	0.71	−45.29	0.1371	5.45	160.14	11
2	6.86	−43.66	0.13554	7.299	161.97	17
3	4.55	−44.86	0.13582	11.077	169.69	18
4	0.74	−41.33	0.13673	4.07	153.61	9
5	2.55	−66.31	0.13617	4.45	164.47	26
6	0.39	−60.26	0.14333	4.89	157.58	34
7	2.05	−45.7	0.14543	3.61	163.36	21
8	0.98	−43.38	0.14087	5.617	159.03	11
9	8.22	−37.44	0.13149	6.58	162.32	14
10	19.58	−35.04	0.13068	4.61	164.13	14
11	17.66	−34.52	0.12936	5.46	156.14	24
12	1.02	−49.98	0.12992	3.25	160.42	9

K_o/w:_ experimental n-octanol-water partition coefficient determined in PBS buffer; δ: dihedral angle determined by the N-oxide function and the α aryl ring; MP2 charge: MP2 calculated charge at the N-oxide α-carbon; μ: DFT calculated dipolar moment; IP: DFT-calculated ionization potential and DOX inhibition: deoxyribose oxidation inhibition.

**Table 2:** Parameter relevance.

Parameter	Coefficient	Coefficient Relevance
K_o/w_	1.29802	1.12175
δ	−1.13928	1.42019
MP2 charge	235.581	0.132496
μ	2.88921	0.762495
IP	−1.36924	0.756433
Constant	129.467	

K_o/w_: experimental n-octanol-water partition coefficient determined in PBS buffer, δ: dihedral angle determined by the N-oxide function and the α-aryl ring; MP2 charge: MP2 calculated charge at the N-oxide α-carbon; μ: DFT calculated dipolar moment; IP: DFT-calculated ionization potential and DOX inhibition: deoxyribose oxidation inhibition.

## Data Availability

Data is contained within the article.
